# Astaxanthin attenuates bisphenol A-induced testicular toxicity in Wistar rats by reducing apoptosis and fibrosis via Bax/Bcl-2 balance and collagen gene expression

**DOI:** 10.17305/bb.2026.13704

**Published:** 2026-02-18

**Authors:** Halime Tuba Canbaz, Mehmet Enes Sozen, Ilknur Cinar Ayan, Hasan Basri Savas, Hilal Arslan, Furkan Adem Canbaz, Gokhan Cuce, Serpil Kalkan

**Affiliations:** 1 Department of Histology and Embryology, Hamidiye Faculty of Medicine, University of Health Sciences, Istanbul, Türkiye; 2 Department of Histology and Embryology, Faculty of Medicine, Alanya Alaaddin Keykubat University, Antalya, Türkiye; 3 Department of Medical Biology, Faculty of Medicine, Necmettin Erbakan University, Konya, Türkiye; 4 Department of Medical Biochemistry, Faculty of Medicine, Mardin Artuklu University, Mardin, Türkiye; 5 Department of Histology and Embryology, Faculty of Medicine, Bakırçay University, İzmir, Türkiye; 6 Department of Pediatric Urology, Sancaktepe Sehit Prof. Dr. İlhan Varank Training and Research Hospital, Istanbul, Türkiye; 7 Department of Histology and Embryology, Faculty of Medicine, Necmettin Erbakan University, Konya, Türkiye

**Keywords:** Astaxanthin, bisphenol A, testicular toxicity, histopathology, oxidative stress, apoptosis.

## Abstract

Bisphenol A (BPA), a synthetic compound widely used in plastic manufacturing, has been shown to cause testicular damage and disrupt spermatogenesis. This study aimed to investigate the potential protective effects of astaxanthin (AST) against BPA-induced testicular injury. Four experimental groups of Wistar Albino rats were established (*n* ═ 8 per group): Control, Sham, BPA, and BPA+AST. At the conclusion of the study, serum samples were analyzed for total antioxidant capacity (TAC), total oxidant status (TOS), oxidative stress index (OSI) [OSI=TOS/TAC], and CRP. Histopathological evaluations included measurements of tubule diameter, Johnsen scoring, and Masson’s trichrome staining. The expression levels of anti-B-cell lymphoma 2 (Bcl-2) and anti-Bcl-2-associated X protein (Bax) were assessed using immunofluorescence (IF) staining and RT-qPCR in testicular tissues. Additionally, tissue collagen (COL1A1, COL3A1) expressions were quantified via RT-qPCR. Results indicated significant increases in TOS, OSI, and CRP levels in the BPA group (*P <* 0.001, *P <* 0.001, and *P ═* 0.042, respectively), while TAC levels remained unchanged (*P ═* 0.119). AST administration did not significantly alter these biochemical parameters. Histopathological analysis revealed decreased Johnsen scores and tubule diameters in the BPA group; however, these metrics improved in the BPA+AST group. IF analysis confirmed that AST restored the pro-apoptotic Bax/Bcl-2 imbalance induced by BPA (*P <* 0.001), although RT-qPCR results indicated that AST normalized only *Bax* expression (*P <* 0.001) while *Bcl-2* levels remained unchanged (*P ═* 0.487). Moreover, *COL1A1* and *COL3A1* were significantly upregulated in the BPA group (*P <* 0.001 for both), and Masson’s trichrome staining corroborated the presence of fibrosis in this group. AST treatment mitigated these fibrotic changes, as evidenced by reductions in gene expression (*P ═* 0.001 for *COL1A1* and *P ═* 0.005 for *COL3A1*) and improvements in Masson’s trichrome staining. In conclusion, this study suggests that AST may confer a protective effect against BPA-induced testicular damage by reducing apoptosis and fibrosis; however, changes in oxidative stress markers did not achieve statistical significance. Furthermore, AST may enhance spermatogenesis.

## Introduction

Bisphenol A (BPA) is a chemical compound utilized in the production of various plastics. Its chemical formula is (CH_3_)_2_C(C_6_H_4_OH)_2_, and it is commonly found in polycarbonates, vinyl ester resins, and epoxy resins. Human exposure to BPA is increasing rapidly due to its extensive use in food and beverage containers, water bottles, medical devices, and can linings [1]. BPA is recognized as an endocrine-disrupting chemical (EDC) because it can mimic endogenous hormones, particularly estrogen [2]. Researches indicate that BPA exposure can lead to reduced sperm count, an increased proportion of abnormal sperm, and disrupted testicular gene expression [3, 4]. Additionally, maternal exposure to BPA during pregnancy has been linked to adverse fetal testicular effects [5, 6]. Studies have shown that oxidative stress induced by BPA plays a significant role in the mechanisms underlying testicular injury [7, 8]. Tissue damage associated with BPA exposure also includes apoptosis and fibrotic remodeling. Fibrosis is characterized by excessive deposition of the extracellular matrix, predominantly comprising type I and III collagens; thus, collagen type I alpha 1 chain (*COL1A1*)/collagen type III alpha 1 chain (*COL3A1*) expression is widely regarded as a molecular marker of active fibrogenesis [9, 10].

Astaxanthin (AST), with the molecular formula C40H52O4, is a naturally occurring xanthophyll carotenoid synthesized by certain microalgae and yeast species [11, 12]. Unlike some other carotenoids, AST cannot be converted to vitamin A in humans; however, its antioxidant and anti-inflammatory properties have led to its application in various diseases. Experimental and clinical studies have demonstrated potential protective effects of AST on liver, kidney, stomach, ocular, dermal, cardiovascular health, and immune modulation [13, 14]. Furthermore, AST has shown promising results in chronic diseases where oxidative stress is a central factor, including diabetes, metabolic syndrome, and neurodegenerative disorders [15, 16].

The present study investigates the efficacy of AST administration in mitigating testicular damage through associated mechanisms.

## Materials and methods

### Animals and experimental design

A total of 32 male Wistar Albino rats were utilized in this experiment. Four groups of eight rats each were established. The rats were 4 months old and weighed between 250 and 300 grams. They were provided with a combination of tap water and standard laboratory food ad libitum. The housing environment maintained a temperature of 24 ± 1^∘^C, a humidity level of 45 ± 5%, and a 12-hour light/dark cycle.

**Group 1 (Control)**: Healthy rats without any treatment or intervention.

**Group 2 (Sham)**: Olive oil was administered daily during the study.

**Group 3 (BPA)**: BPA dissolved in olive oil was administered daily during the study.

**Group 4 (BPA+AST)**: Both BPA and AST, dissolved in olive oil, were administered daily during the study.

All substances, including olive oil, BPA, and AST, were administered via oral gavage. The duration of the experiment was 2 weeks. The doses of BPA (250 mg/kg) and AST (20 mg/kg) were determined based on existing literature [17, 18]. BPA (purity: ≥99%, Chemical Abstracts Service (CAS) No. 80-05-7, Sigma Aldrich) and AST (purity: 98%, CAS No. 472-61-7, Sigma Aldrich) were freshly prepared daily immediately before administration. BPA was dissolved in olive oil at a concentration of 100 mg/mL, while AST was dissolved in olive oil at a concentration of 8 mg/mL. For both compounds, the doses corresponded to a gavage volume of 2.5 mL/kg, resulting in an administered volume of approximately 0.625–0.75 mL per animal based on body weight. BPA was administered first, followed by AST after a 2-hour interval.

On the fourteenth day of the experiment, anesthesia was induced via intraperitoneal injection of 50 mg/kg ketamine HCl and 10 mg/kg xylazine HCl. Blood samples were collected through cardiac puncture, and all rats were subsequently sacrificed via exsanguination. Testicular tissues were macroscopically examined for gross abnormalities (discoloration, edema, hemorrhage, focal lesions, or marked atrophy) prior to tissue collection. No overt macroscopic abnormalities were noted.

**Table 1:** Primer sequences utilized in RT-qPCR analysis

**Gene**	**Forward primer (5′-3′)**	**Reverse primer (5′-3′)**
Bax	GATGGCCTCCTTTCCTACTTC	CTTCTTCCAGATGGTGAGTGAG
Bcl-2	GGAGGATTGTGGCCTTCTTT	GTCATCCACAGAGCGATGTT
COL1A1	CCAATGGTGCTCCTGGTATT	GTTCACCACTGTTGCCTTTG
COL3A1	GTGTGATGATGAGCCACTAGAC	TGACAGGAGCAGGTGTAGAA
GAPDH	GCATTGCAGAGGATGGTAGAG	GCGGGAGAAGAAAGTCATGATTAG

**Abbreviations:** Bax: Bcl-2-associated X protein; Bcl-2: B-cell lymphoma 2; COL1A1: Collagen type I alpha 1 chain; COL3A1: Collagen type III alpha 1 chain; GAPDH: Glyceraldehyde-3-phosphate dehydrogenase; RT-qPCR: Reverse transcription quantitative polymerase chain reaction.

### Biochemical analysis

Blood samples were placed into biochemistry tubes containing gel, then centrifuged at 1500 g for 10 min to obtain serum. The sera were aliquoted and stored at –80 ^∘^C in Eppendorf tubes until analysis. Serum samples were sent to the biochemistry laboratory labeled without group identifiers and analyzed blindly by the investigator. Prior to examination, each blood sample was thawed and acclimatized to room temperature. Each serum sample was vortexed to ensure homogeneity before biochemical analyses.

Total antioxidant capacity (TAC) and total oxidant level (TOS) in serum samples were analyzed using fully automated colorimetric methods (Rel Assay Diagnostics, Turkey). TAC levels were expressed in micromolar Trolox equivalent (µmol Trolox eq/L) [19]. TOS levels were reported as micromolar hydrogen peroxide equivalent (µmol H_2_O_2_ eq/L) [20]. Subsequently, the oxidative stress index (OSI) was calculated using the formula: OSI = TOS/TAC. Measurement of serum C-reactive protein (CRP) was performed using the Abbott Architect c8000 analyzer and corresponding kits (Abbott, Illinois, USA). CRP levels were reported in mg/L.

### Histopathological analysis

Testicular tissues were examined macroscopically and stored in 10% formalin. After processing, tissues were embedded in paraffin and 5 µm-thick sections were taken from the paraffin blocks. Histopathological examination included Masson’s trichrome staining to assess fibrosis, and hematoxylin-eosin (H&E) staining for Johnsen scoring and tubule diameter assessment. Histopathological investigation was conducted using a double-blind methodology with a Zeiss Lab.A1 light microscope.

Sections underwent xylene treatment (three times for 20 min each) and were subjected to a descending sequence of alcohol concentrations (100%, 96%, and 70%) for deparaffinization. Specimens were subsequently stained using Harris hematoxylin (Bio Optica 05–06004) for 4 min and counterstained with 1% aqueous eosin (Bio Optica 05–10007) for 2 min. H&E staining was performed to evaluate general histological structure and spermatogenesis. The Johnsen testicular scoring system was employed for quantitative assessment of spermatogenesis [21], allowing the evaluation of each seminiferous tubule on a scale from 1 (total loss of germ cells) to 10 (normal spermatogenesis) based on the configuration of germinal epithelial cells. Average scores were determined by analyzing a minimum of 20 randomly selected seminiferous tubules from each specimen. Additionally, tubule diameters were measured in ten cross-sections of each specimen.

Masson’s trichrome staining was utilized to evaluate the augmentation of connective tissue and fibrotic alterations in testicular tissues using the ChemBio staining kit CB6595.0100 [22].

### Immunofluorescence (IF) analysis

Sections were mounted on lysine-coated slides. After deparaffinization and rehydration, antigen retrieval and endogenous peroxidase blocking were performed. Super Block (ScyTek Laboratories, Logan, UT) was also applied to mitigate nonspecific antigen binding. The sections were incubated overnight with primary antibodies: Anti-B-cell lymphoma 2 (Bcl-2) (1:500; mouse monoclonal antibody, sc-7382, Santa Cruz Biotechnology) and Anti-Bcl-2-associated X protein (Bax) (1:100; mouse monoclonal antibody, sc-23959, Santa Cruz Biotechnology). Following this, Goat Anti-Mouse IgG heavy and light chains (H&L) (Alexa Fluor^®^ 488) ab150113 was applied for 1 hour at room temperature. Slides were then covered with a medium containing phosphate-buffered saline (PBS), glycerine, and Hoechst 33342 (ThermoFisher Scientific) and observed using a Zeiss Axio microscope equipped with a fluorescence attachment, utilizing Kameram software [23]. Ten randomly selected fields from six sections per animal were analyzed using ImageJ software, and an individual mean value for each animal was obtained. These mean values were used for statistical analysis, treating each animal as the experimental unit (*n* ═ 8 per group). Mean intensity was assessed following background subtraction and normalization to the tissue/DAPI area [24].

### RT-qPCR analyses

In the RT-qPCR analysis, all equipment was sterilized with 70% alcohol prior to dissection to minimize RNA degradation. Low-temperature-resistant, nuclease-free cryovial tubes (2 mL) were labeled before dissection, and the experiment commenced after preparing liquid nitrogen. Immediately following euthanasia, testicular tissues were rapidly excised and separated from blood and adipose tissues (maximum time: 1–2 min). After a brief wash with RNase-free PBS, the tissues were cut into pieces and promptly placed in cryovial tubes, which were subsequently immersed in liquid nitrogen. The testicular tissues were stored at –80 ^∘^C until RNA isolation.

To assess gene expression levels of *Bax, Bcl-2*, *COL1A1*, and *COL3A1* at the mRNA level, total RNA was extracted from the testicular tissues using GeneAll RiboEX (301–001). Due to the fibrous nature of testicular tissue, each sample was homogenized in 1 mL of RiboEX reagent using a homogenizer to ensure complete degradation during RNA isolation. After phase separation with chloroform and RNA precipitation, the RNA pellet was washed with ethanol and dissolved in RNase-free water under slow pipetting conditions. The quality and quantity of the isolated RNA were assessed using a Nanodrop spectrophotometer at 260 and 280 nm wavelengths, yielding a 260/280 ratio of approximately 1.9–2.2 and quantities of around 2000–3000 ng. To eliminate DNA contamination, DNase I enzyme (Thermo Fisher Scientific, #EN0521) was applied to all RNA samples at a concentration of 1 µg. The iScript™ cDNA synthesis kit (BIO-RAD, 170–8891) was employed to convert the extracted RNA into cDNA. A No-RT (No Reverse Transcription) reaction was concurrently set up under identical conditions but without the reverse transcriptase enzyme to serve as a control. This control was necessary to determine whether the signal observed in RT-qPCR was derived from cDNA (i.e., RNA origin) or from genomic DNA (gDNA) contamination.

**Figure 1.**
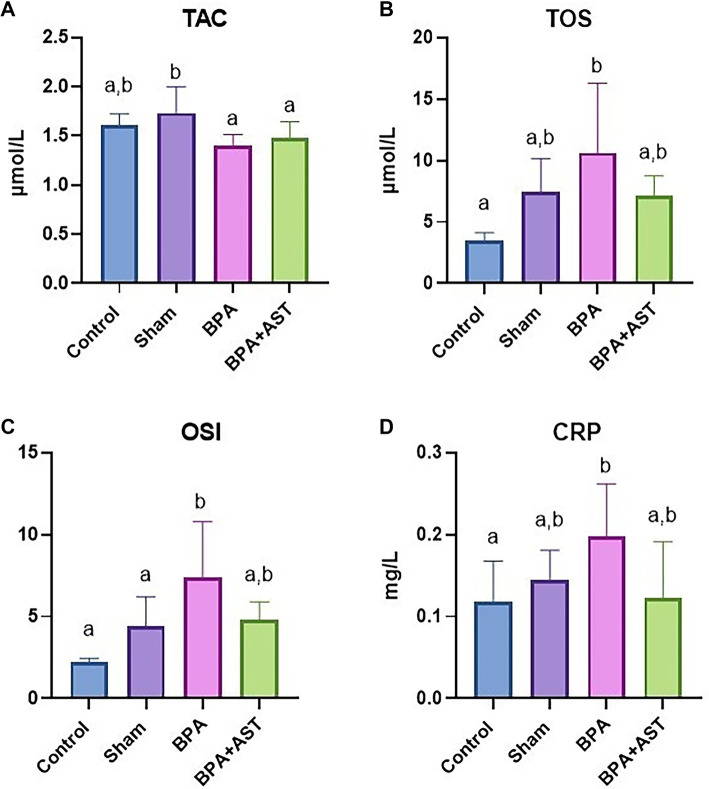
**Comparison of biochemical parameters among groups.** (A) TAC; (B) TOS; (C) OSI (OSI = TOS/TAC); (D) CRP levels of groups. Serum TOS and OSI were increased in the BPA group versus Control (both *P <* 0.001), and CRP was elevated versus Control (*P ═* 0.042), whereas TAC did not differ versus Control (*P ═* 0.119). In the BPA+AST group, TOS, OSI, and CRP were numerically lower than in the BPA group, but these reductions did not reach statistical significance (*P ═* 0.155, *P ═* 0.069, and *P ═* 0.056, respectively). There is statistically significant difference between groups not sharing the same letter (*P <* 0.05). Data are presented as mean ± SD. **Abbreviations:** AST: Astaxanthin; BPA: Bisphenol A; CRP: C-reactive protein; OSI: Oxidative stress index; SD: Standard deviation; TAC: Total antioxidant capacity; TOS: Total oxidant status.

All cDNA samples were diluted to a standard concentration of 1 µg with nuclease-free water. Additionally, a No Template Control (NTC) reaction was prepared prior to RT-qPCR. Primer specificity was confirmed by melt curve analysis post-amplification, with a single, sharp peak indicating specific amplification (Figure S1). NTC samples were also analyzed to rule out non-specific products and primer-dimer formation. These validation steps confirmed the suitability of the primers for qPCR analyses. Primers for the target genes (*Bax, Bcl-2, COL1A1, COL3A*1) and reference genes (*GAPDH*) were designed using the Integrated DNA Technologies (IDT) PrimerQuest program (Table 1). Real-time qPCR was conducted using a 5× HOT FIREPol^®^ EvaGreen^®^ RT-qPCR Master Mix Plus (ROX) (Solis BioDyne) and the BIO-RAD CFX Connect™ Real-Time System under the following conditions: 12 min at 95^∘^C, followed by 40 cycles of 95^∘^C for 15 s, 60^∘^C for 20 s, and 72^∘^C for 20 s. RT-qPCR reactions were performed in a single well for each biological replicate, with each replicate corresponding to an independent animal (*n* ═ 8 per group). Given the high number of independent biological replicates per group (*n* ═ 8), single technical replicate measurements were deemed sufficient. Gene expression levels were normalized to the reference gene [13]. Tissue samples were labeled to ensure the researcher conducting the RT-qPCR analysis was blinded to the group identity, facilitating an unbiased assessment. To minimize inter-assay variability and maximize technical consistency, all samples for each target gene and their corresponding reference gene were analyzed within a single 96-well plate using the BIO-RAD CFX96 Connect™ Real-Time System. This high degree of standardization across experimental groups (*n* ═ 8 per group) prioritized biological replicates, allowing for the simultaneous quantification of 64 reactions (32 target and 32 reference) in a single run, thus eliminating plate-to-plate variation, which is often a more significant source of error than intra-assay technical replication.

### Statistical analysis

Statistical evaluations were conducted using GraphPad Prism (version 8.4.2). Continuous variables were presented as means ± SDs. The normality of data across groups was assessed using the Shapiro-Wilk test, histograms, and Q-Q plots. One-way ANOVA was utilized to analyze normally distributed numerical data among groups. Homogeneity of variances was verified with the Brown–Forsythe test, followed by Tukey’s post hoc test for pairwise comparisons. Primary endpoints reflecting core tissue-level effects—spermatogenesis, assessed via Johnsen scoring, and apoptosis, evaluated through immunofluorescence of Bax expression—were predefined, with multiplicity controlled using the Holm–Bonferroni procedure. All additional oxidative stress markers and molecular targets were categorized as exploratory and interpreted accordingly. After normalization to the reference gene, relative gene expression levels were calculated using the 2^−ΔΔCt^ method. Primer specificity was confirmed through melt curve analysis, ensuring a single, specific amplification product for each primer pair.

Both ΔCt and fold change values (2^−ΔΔCt^) were statistically analyzed using one-way ANOVA followed by Tukey’s post hoc test for comparisons between experimental groups. A *P* value of less than 0.05 was considered indicative of statistical significance.

**Figure 2.**
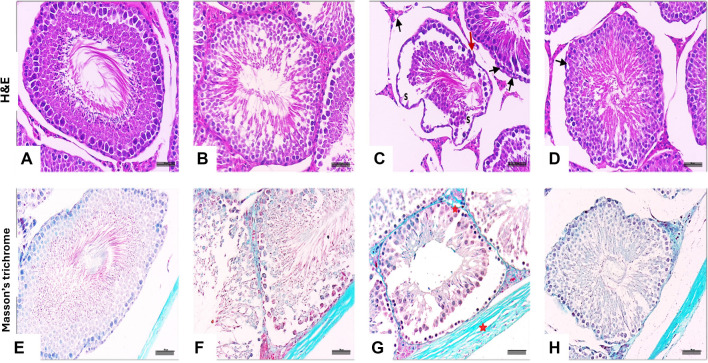
**H&E and Masson’s trichrome staining of testicular specimens belonging to groups.** Representative H&E (A–D) and Masson’s trichrome (E–H) sections from the Control (A, E), Sham (B, F), BPA (C, G), and BPA+AST (D, H) groups. The Control and Sham groups show normal testicular organization with orderly seminiferous tubules, intact germinal epithelium and interstitial compartments, and spermatogenic cells at multiple differentiation stages with a continuous basement membrane. In the BPA group, seminiferous tubules are dysmorphic, shrunken, and disorganized with compromised epithelial integrity and interstitial deterioration, characterized by germ-cell detachment/degeneration with sloughing (s), cytoplasmic vacuolization (black arrows), and basement membrane disruption (red arrow). Masson’s trichrome highlights fibrotic remodeling in the BPA group, including thickening of the tunica albuginea/basement membrane and increased collagen accumulation (red star). In the BPA+AST group, testicular architecture is relatively preserved, showing only minimal degenerative changes (including focal vacuolization) and an overall reduction of fibrotic thickening compared with BPA. Scale bars, 50 µm. **Abbreviations:** AST: Astaxanthin; BPA: Bisphenol A; H&E: Hematoxylin and eosin.

**Figure 3.**
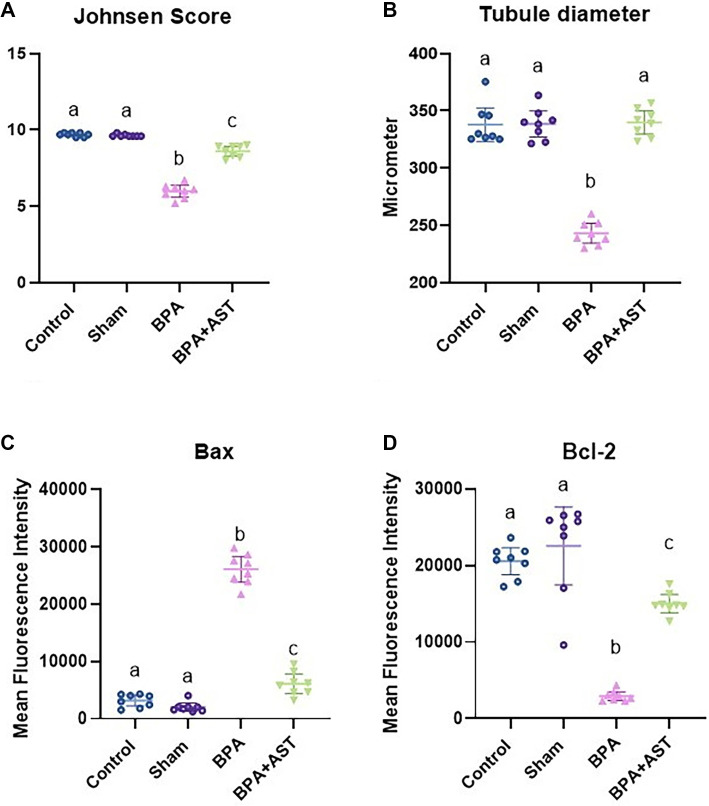
**Histopathological assessment scores of experimental groups.** (A) Johnsen scores; (B) tubule diameters; (C) Bax; and (D) Bcl-2 expression IF intensity of groups (Control, Sham, BPA, and BPA+AST; *n* ═ 8/group). BPA exposure markedly impaired spermatogenesis, reflected by reduced Johnsen scores versus Control (*P <* 0.001), while AST significantly ameliorated this disruption in the BPA+AST group versus BPA (*P <* 0.001). Seminiferous tubule diameter was decreased in the BPA group and restored toward Control/Sham values by AST. IF quantification demonstrated a BPA-induced pro-apoptotic shift, with increased Bax and decreased Bcl-2 versus Control (both *P <* 0.001), whereas AST reversed these changes (both *P <* 0.001 vs BPA); for Bax, the adjusted *P* value remained <0.001 after Holm–Bonferroni correction. There is statistically significant difference between groups not sharing the same letter (*P <* 0.05). Data are shown as individual data points with mean and 95% CI. **Abbreviations:** AST: Astaxanthin; Bax: Bcl-2-associated X protein; Bcl-2: B-cell lymphoma 2; BPA: Bisphenol A; CI: Confidence interval; IF: Immunofluorescence.

### Ethical statement

This study was approved by the Animal Experiments Local Ethics Committee at Necmettin Erbakan University (Decision date: 30.12.2022, Decision number: 2022-072). The research adhered to institutional protocols and international standards for the care and use of laboratory animals.

## Results

### Biochemical analyses

The mean TOS level was significantly higher in the BPA group (11.0 ± 5.60 µmol/L) compared to the control group (3.5 ± 0.59 µmol/L) (*P <* 0.001). No significant difference was observed between the sham group (7.5 ± 2.70 µmol/L) and the control group regarding TOS levels (*P ═* 0.091). Although the BPA+AST group (7.1 ± 1.60 µmol/L) exhibited reduced TOS levels compared to the BPA group, this difference did not achieve statistical significance (*P ═* 0.155).

The mean TAC levels for the control and sham groups were comparable, measuring 1.6 ± 0.12 µmol/L and 1.7 ± 0.27 µmol/L, respectively (*P ═* 0.483). The BPA group demonstrated a lower mean TAC level of 1.4 ± 0.12 µmol/L compared to the control group; however, this difference was not statistically significant (*P ═* 0.119). In the BPA+AST group, AST application appeared to counteract the reduction, resulting in a mean TAC level of 1.5 ± 0.17 µmol/L, although this difference also lacked statistical significance (*P ═* 0.483).

BPA treatment resulted in a significant increase in OSI levels compared to the control group, with values of 7.40 ± 3.38 vs. 2.19 ± 0.27, respectively (*P <* 0.001). No significant difference was noted between the control and sham groups, which measured 4.44 ± 1.77 (*P ═* 0.133). The mean OSI level for the BPA+AST group was 4.83 ± 1.08. While AST application indicated a decrease in OSI levels elevated by BPA treatment, this reduction was not statistically significant (*P ═* 0.069).

Serum CRP levels in the sham group (0.145 ± 0.036 mg/L) did not differ significantly from the control group (0.119 ± 0.049 mg/L) (*P ═* 0.786). The BPA group exhibited elevated CRP levels (0.198 ± 0.064 mg/L) compared to the control group (*P ═* 0.042). Conversely, CRP levels in the BPA+AST group were numerically lower (0.123 ± 0.069 mg/L) than in the BPA group; however, this reduction was not statistically significant (*P ═* 0.056). The biochemical findings of the study are illustrated in Figure 1.

### Histopathological evaluation

In both the control and sham groups, the seminiferous tubules exhibited an orderly structure, with intact epithelium and interstitial areas. Various stages of spermatogenic cells were observed, and the basement membrane remained intact. In contrast, the BPA group displayed shrunken and disorganized seminiferous tubules, with compromised epithelial integrity. Figure 2 illustrates the detachment and degeneration of germ cells, as well as the deterioration of interstitial areas characterized by sloughing and vacuolization. Notably, the basement membrane integrity was significantly compromised in the BPA group. The BPA+AST group exhibited relatively better histological conditions, though it still showed slight degenerative changes, including cytoplasmic vacuolization in the seminiferous tubules.

Statistical analysis revealed significant differences among groups in terms of Johnsen scoring (*P <* 0.001 for both raw and adjusted *P* values, Holm–Bonferroni correction). The mean Johnsen score for the control group was 9.69 ± 0.12, with nearly all rats demonstrating full spermatogenesis. The sham group had a mean Johnsen score of 9.64 ± 0.07, which was statistically similar to that of the control group (*P ═* 0.989). The BPA treatment adversely affected spermatogenesis, resulting in a reduced Johnsen score of 5.98 ± 0.47 compared to the control (*P <* 0.001). AST administration significantly mitigated this disruption (8.60 ± 0.39), as indicated by statistical analysis (*P <* 0.001) (Figure 3). Additionally, tubule diameters were significantly decreased in the BPA group compared to both the control and sham groups, while AST treatment reversed this reduction (Figure 3).

Masson’s trichrome staining was utilized to visualize collagen fibers. In both the control and sham groups, the tunica albuginea and basement membranes appeared normal-sized, whereas both structures were thickened in the BPA group. The BPA+AST group demonstrated a similar appearance of the basement membrane and tunica albuginea as seen in the control and sham groups (Figure 2).

### IF evaluation

Significant differences among groups were noted in Bax levels (*P <* 0.001). No significant differences were observed between the control and sham groups regarding Bax, a pro-apoptotic factor (*P ═* 0.603). The BPA group exhibited a significantly elevated expression of Bax compared to the control (*P <* 0.001). AST administration effectively reversed the BPA-induced increase in Bax levels (*P <* 0.001). Adjusted *P* values for Bax, considering multiplicity across the two primary endpoints, were <0.001 (Holm–Bonferroni correction).

Bcl-2, an anti-apoptotic factor, showed no significant difference between the control and sham groups (*P ═* 0.627).

However, Bcl-2 expression was reduced in the BPA group compared to the control, but elevated in the BPA+AST group (*P <* 0.001 for both comparisons). These findings are illustrated in Figure 3.

**Figure 4.**
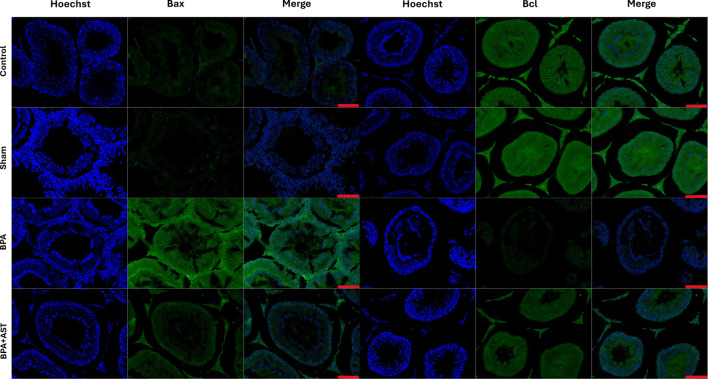
**Immunofluorescence staining of Bax and Bcl-2 among groups.** Representative IF micrographs of testicular sections from the Control, Sham, BPA, and BPA+AST groups showing nuclei counterstained with Hoechst (blue) and immunoreactivity for Bax or Bcl-2 (green). Consistent with the quantitative IF findings, BPA exposure increased Bax staining and reduced Bcl-2 staining relative to Control/Sham, whereas AST co-treatment attenuated Bax signal and restored Bcl-2 immunoreactivity. All “Merge” panels show the overlay of Hoechst and antibody channels; scale bars, 100 µm. **Abbreviations:** AST: Astaxanthin; Bax: Bcl-2-associated X protein; Bcl-2: B-cell lymphoma 2; BPA: Bisphenol A; IF: Immunofluorescence.

Figure 4 presents immunofluorescence staining, including Hoechst staining of nuclei in blue and antibody staining intensities in green.

### Analysis of gene expression at the mRNA level

The effects of BPA and BPA combined with AST treatment on the mRNA expression of genes related to apoptosis (*Bax, Bcl-2*) and collagen (*COL1A1, COL3A1*) in testicular tissues were assessed using RT-qPCR. The results demonstrated that *Bax* gene expression increased by 3.19-fold (*P <* 0.001; one-way ANOVA, post hoc Tukey test; F (3,20) ═ 21.24) in the BPA group compared to the control group, and by 2.81-fold (*P <* 0.001; one-way ANOVA, post hoc Tukey test; F (3,20) ═ 21.24) relative to the sham group. Furthermore, a 2.16-fold (*P <* 0.001; one-way ANOVA, post hoc Tukey test; F (3,20) ═ 21.24) decrease in *Bax* mRNA expression was observed in the BPA+AST group compared to the BPA group.

In evaluating *Bcl-2* mRNA expression, a decrease of 2.17-fold (*P ═* 0.025; one-way ANOVA, post hoc Tukey test; F (3,20) ═ 4.639) was noted in the BPA group relative to the control group, and a 2.19-fold decrease (*P ═* 0.022; one-way ANOVA, post hoc Tukey test; F (3,20) ═ 4.639) compared to the sham group. The BPA+AST group demonstrated a 1.54-fold increase in *Bcl-2* gene expression compared to the BPA group; however, this improvement was not statistically significant (*P ═* 0.487; one-way ANOVA, post hoc Tukey test; F (3,20) ═ 4.639).

Analysis of *COL1A1* mRNA expression revealed a 2.72-fold (*P <* 0.001; one-way ANOVA, post hoc Tukey test; F (3,20) ═12.32) and a 2.85-fold (*P <* 0.001; one-way ANOVA, post hoc Tukey test; F (3,20) ═12.32) increase in the BPA group compared to the control and sham groups, respectively. In the BPA+AST group, *COL1A1* gene expression was reduced by 2.25-fold (*P ═* 0.001; one-way ANOVA, post hoc Tukey test; F (3,20) ═ 12.32) compared to the BPA group.

**Figure 5.**
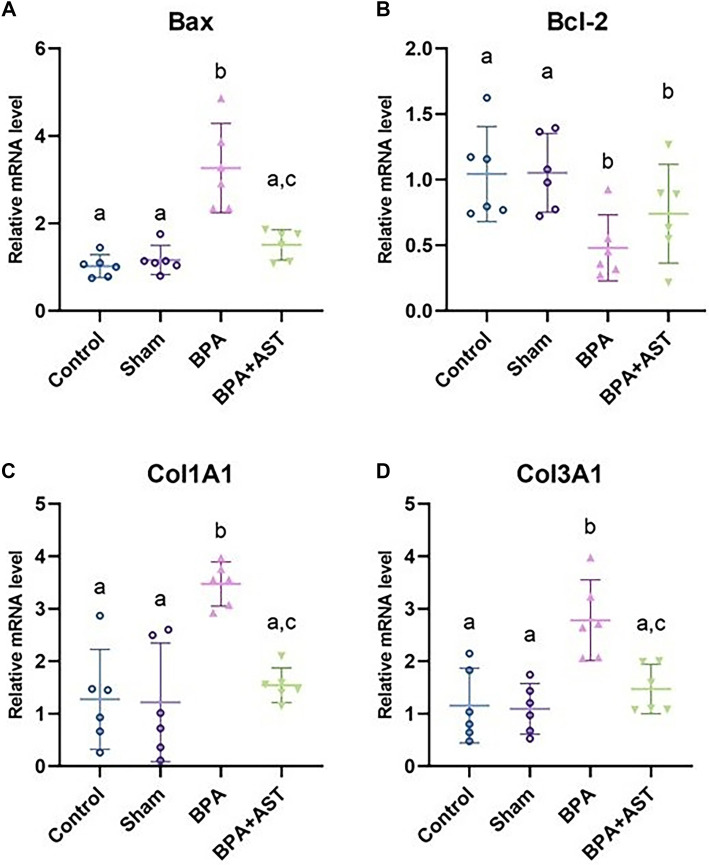
**RT-qPCR analysis of the mRNA expression level among groups.** Relative mRNA levels of (A) Bax, (B) Bcl-2, (C) Col1A1, and (D) Col3A1 in testicular tissues from the Control, Sham, BPA, and BPA+AST groups (*n* ═ 8/group). BPA exposure increased Bax expression versus Control and Sham (*P <* 0.001 for both) and decreased Bcl-2 expression versus Control and Sham (*P ═* 0.025 and *P ═* 0.022, respectively). BPA also upregulated Col1A1 and Col3A1 versus Control and Sham (*P <* 0.001 for all comparisons). AST co-treatment reduced Bax expression versus BPA (*P <* 0.001) but did not significantly restore Bcl-2 at the mRNA level (*P ═* 0.487 vs BPA). AST significantly attenuated BPA-induced collagen gene upregulation (Col1A1: *P ═* 0.001; Col3A1: *P ═* 0.005 vs BPA). There is statistically significant difference between groups not sharing the same letter (*P <* 0.05). Data are shown as individual data points with mean and 95% confidence intervals (95% CI). **Abbreviations:** AST: Astaxanthin; Bax: Bcl-2-associated X protein; Bcl-2: B-cell lymphoma 2; BPA: Bisphenol A; CI: Confidence interval; Col1A1: Collagen type I alpha 1 chain; Col3A1: Collagen type III alpha 1 chain; mRNA: Messenger RNA; RT-qPCR: Reverse transcription quantitative polymerase chain reaction.

Comparative analysis of the BPA group with the control and sham groups indicated a 2.41-fold (*P <* 0.001; one-way ANOVA, post hoc Tukey test; F (3,20) ═ 10.59) and a 2.54-fold (*P <* 0.001; one-way ANOVA, post hoc Tukey test; F (3,20) ═ 10.59) increase in mRNA expression levels of the *COL3A1* gene, respectively. In contrast, the BPA+AST group showed a reduction of 1.88-fold (*P ═* 0.005; one-way ANOVA, post hoc Tukey test; F (3,20) ═ 10.59) in *COL3A1* mRNA expression compared to the BPA group. Findings related to gene expression at the mRNA level are illustrated in Figure 5.

## Discussion

This study evaluated the protective effect of AST in a testicular injury model induced by BPA exposure, assessing histological, IF, biochemical, and molecular parameters. Previous research established that a BPA dosage of 50 mg/kg is the lowest amount producing side effects [2]. Additionally, testicular damage was found to be more pronounced at dosages of 90 mg/kg and 270 mg/kg, with the most significant effects observed at 270 mg/kg [7]. Consistent with existing literature, our study demonstrated that BPA administration induced oxidative stress parameters, apoptosis markers, and fibrosis-related gene expression, thereby leading to testicular damage.

The role of oxidative stress in the mechanism underlying BPA-induced testicular toxicity is well-documented. Research has shown decreased TAC levels in the testicular tissue of rats exposed to BPA [25, 26]. Ling et al. indicated that this decline is associated with reduced levels of the nuclear factor erythroid 2–related factor 2 (Nrf2) protein and its target genes, *NQO1* and glutathione peroxidase 1 (*GPx-1*) [27]. Furthermore, BPA exposure was linked to increased levels of malondialdehyde (MDA) in testicular tissue, alongside reduced glutathione (GSH) levels and activities of GSH-peroxidase (GSH-PX) and superoxide dismutase (SOD) [3, 28]. The efficacy of various antioxidants in mitigating testicular toxicity through this mechanism has been investigated. Apilarnil has shown effectiveness in reversing elevated tissue levels of MDA and decreasing GSH levels [29]. Similarly, glutamine and coenzyme Q have been reported to effectively reduce BPA-associated testicular damage by enhancing levels of tissue MDA, catalase (CAT), and SOD [30, 31]. AST has been noted for alleviating testicular damage resulting from cadmium toxicity due to its antioxidant properties and its ability to improve tissue TAC levels [32]. In the present study, while BPA elevated oxidative stress, the increase in oxidative stress parameters following AST administration was not statistically significant. We posit that the lack of significant changes in TOS, TAC, and OSI values, despite observed improvements in histological and molecular evaluations—can be attributed to the assessment of oxidative stress in serum rather than in tissue. Additionally, the study may have been underpowered to detect subtle differences; thus, non-significant findings—particularly concerning TAC, TOS, and OSI—should be interpreted as inconclusive rather than definitive evidence of no effect.

Johnsen scoring is a widely utilized histological evaluation technique for assessing the extent of testicular damage [33]. One investigation indicated that BPA exposure adversely affects spermatogenesis within seminiferous tubules [25]. Shetty et al. reported that Johnsen scores decline with BPA exposure but increase with the administration of mitochondria-targeted antioxidant agents [34]. The current study corroborates existing literature by histologically demonstrating testicular damage induced by BPA using the Johnsen score. Furthermore, AST administration showed improvements in seminiferous tubule architecture and spermatogenesis. The diameters of the tubules also varied in association with these effects. The BPA group exhibited decreased tubule diameters as a result of BPA’s destructive impact on the seminiferous epithelium. In the BPA+AST group, tubule diameters were comparable to those in the control and sham groups, suggesting that AST mitigates the detrimental effects of BPA on tubule diameter.

Bax and Bcl-2 are proteins involved in the regulation of apoptosis, with Bax promoting and Bcl-2 inhibiting apoptotic processes [35, 36]. In the context of BPA-related testicular toxicity, oxidative stress has been shown to activate apoptosis pathways and shift the Bax/Bcl-2 balance toward a pro-apoptotic state [37–39]. Antioxidants like hesperidin and selenium have demonstrated efficacy in rebalancing this pro-apoptotic equilibrium [40, 41]. Our study revealed that BPA exposure increases apoptosis by elevating Bax levels and reducing Bcl-2 levels. Conversely, AST administration appears to prevent this apoptotic decline. IF analysis indicated an increase in Bcl-2 protein levels following AST treatment, supporting its hypothesized anti-apoptotic effects at the cellular level. Although quantitative PCR indicated an increase in *Bcl-2* mRNA expression that did not reach statistical significance, this discrepancy may reflect regulatory mechanisms predominantly occurring at the protein level rather than the transcriptional level. The stability of the Bcl-2 protein may enhance cell survival without substantial changes in gene expression. Therefore, the observed increase in Bcl-2 protein expression is physiologically relevant and aligns with the intended anti-apoptotic function of the therapeutic agent.

BPA has been shown to induce fibrosis in various organs. Increased mRNA expression of pro-fibrotic regulators *Col1A1* and *Col3A1* has been documented in cardiac tissue [10]. Gültekin et al. demonstrated that the expression of *Col1A1* and *Col3A1* elevated in kidney tissue following BPA administration was reduced with AST treatment [42]. Zhao et al. reported that collagen type I alpha 2 chain (*Col1A2*) expression in testicular tissue increased with BPA exposure, whereas administration of syringin mitigated this effect [43]. Our study’s findings align with existing literature, revealing that BPA elevated *COL1A1/COL3A1* expression, indicating the activation of a pro-fibrotic transcriptional pathway. While collagen mRNA levels do not directly reflect mature collagen deposition, the up-regulation of type I/III collagen genes is often regarded as an early molecular indicator of fibrogenesis that may precede visible tissue fibrosis. AST administration effectively protects testicular tissue through its antifibrotic properties.

Serum CRP levels, a marker of inflammation, have been shown to increase in cases of BPA-related testicular injury [44]. Kohandel et al. demonstrated that AST reduces serum CRP levels and may be beneficial in treating multiple diseases due to its anti-inflammatory properties [13]. This study indicated that serum CRP levels rose with BPA exposure, while AST administration led to decreased serum CRP levels, although this was not statistically significant.

This study acknowledges several limitations. Firstly, the number of animals and experimental groups was constrained by institutional conditions and resources. A significant limitation is the absence of a separate group receiving solely AST treatment. Secondly, the tissue concentrations of biochemical parameters, especially oxidative stress indicators, were not measured, preventing direct quantification of local testicular redox status. Additionally, the study duration limits the ability to draw long-term conclusions. Although testicular damage was assessed through various parameters, including biochemical, histopathological, immunofluorescence, and RT-qPCR techniques, fertility outcomes were not evaluated. Lastly, given that multiple outcomes were assessed concurrently, the potential for random findings cannot be overlooked; hence, these biomarkers should be regarded as supportive/exploratory, with non-significant results interpreted as inconclusive.

## Conclusion

AST administration has been shown to reduce apoptosis and fibrosis associated with BPA-induced testicular damage; however, AST did not result in statistically significant changes in serum oxidative stress markers. Furthermore, AST appears to play a protective role in spermatogenesis.

## Supplemental data

Supplemental data are available at the following link: https://www.bjbms.org/ojs/index.php/bjbms/article/view/13704/4135.

## Data Availability

The data that support the findings of this study are available from the corresponding author upon reasonable request.
